# Supplementary material for: Gut Microbiota in Diagnosis, Therapy and Prognosis of Cholangiocarcinoma and Gallbladder Carcinoma—A Scoping Review
Source: Microorganisms. 2023 Sep 21;11(9):2363. doi: 10.3390/microorganisms11092363 (PMC10538110; doi:10.3390/microorganisms11092363)
Supplement: Supplementary file 1 [file microorganisms-11-02363-s001.zip › microorganisms-2543124-supplementary.pdf]

Supplementary Material – Methods focusing on microbiota analysis of all included studies:

(quoted from the responding original publication)

Caygill et al. [55] did not provide information about bacterial analysis. Nagaraja et al. [43] performed a systematic review, which is why we waived to report methods in detail.

Koshiol et al. [44]:

"All samples […] were analyzed by real-time PCR (qPCR) to detect S. Typhi, employing primers to amplify fliC, which encodes the S. Typhi Phase 1 flagellin subunit (Hd) […]. The analytical sensitivity of the PCR was 14.5 S. Typhi genomes per qPCR reaction (data not shown). Conversely, DNA extracted from clinical isolates of S. Typhimurium, S. Choleraesuis, S. Paratyphi A, or S. Enteritidis did not amplify. Gel-based PCR was used to detect Salmonella species, utilizing primers that amplify invA encoding a Salmonella Pathogenicity Island 1 protein required for invasion of epithelial cells […]). PCR products for Salmonella species were visualized on agarose gels, 1.5%. DNA extracted from a clinical isolate of S. Typhi was used as the positive control in qPCR and gel-based PCR tests. PCR-grade water was used as the negative control in all tests. Human RNAse P gene was used as the internal quality control to ensure proper DNA extraction and the absence of PCR inhibitors […]."

Miyabe et al. [45]:

"Genomic DNA extraction, next-generation sequencing, and bioinformatics processing of the sequenced reads are fully described in the Supplementary Materials. Both bile and stool 16S rDNA amplicons were sequenced on one lane of a MiSeq (Illumina Inc., San Diego, CA, USA). Pre-processed sequence files were then analyzed via the hybrid de novo bioinformatics pipeline [11] with the default parameter settings to obtain the operational taxonomic unit (OTU) table. OTUs were assigned taxonomy using the Ribosomal Database Project (RDP) classifier trained on the Greengenes database (v13.5) and a phylogenetic tree was built based on FastTree [12]. Due to their low bacterial content and the inability to reliably characterize their bacterial profiles and exclude the influence of environmental contamination, samples with fewer than 2000 reads were excluded from the analyses of the microbiome profiles at QC. After QC of the study samples, 3,751,425 reads remained (median: 23,652 reads per sample, range: 2220 to 58,443 reads per sample, lower and upper quartiles: 16,853 and 30,939 reads per sample). The OTUs belong to 18 phyla, 98 families and 162 genera based on the RDP classifier on the Greengenes database. […] For the microbiome analyses, sources of variability in the dataset were identified by testing associations between the overall microbiota composition and various clinical variables using the PERMANOVA-based omnibus test […]. Detailed comparative analyses of the bile and stool microbiota from controls vs. PSC, PSC vs. CCA with PSC (CCA w PSC), and controls vs. CCA without PSC (CCA wo PSC), were performed at -diversity, -diversity and taxa abundances. To test whether the bile and stool samples from the same subject were similar, a distance-based permutation approach was used. […]"

Ma et al. [46]:

"All participants' faecal samples were frozen and stored in a −80°C refrigerator after collection immediately. In order to reduce the effect of collecting samples with different faecal locations, we collected the middle faeces of all participants as samples. The genomic DNA of the gut microbiome was extracted by the EZNA® Stool DNA Kit (D4015, Omega, Inc.). The DNA extraction process was performed using ultra-pure water to avoid false-positive PCR results. High-throughput V3-V4 regions of 16s rRNA identified gut microbiome classification and composition in faecal samples.

Subsequently, the amplification of the V3-V4 high-throughput region of 16s rRNA via PCR reaction (Thermo Scientific® Phusion High-Fidelity Hot start flex 2X Master Mix [New England Biolabs]) with primers 341F (5'-CCTACGGGNGGCWGCAG-3′) and 805R (5'-GACTACHVGGGTATCTAATCC-3′). The PCR products were purified with AMPure XT beads (Beckman Coulter Genomics) and quantified with Qubit (Invitrogen, USA). The amplicon pools were prepared for sequencing by the Agilent 2100 Bioanalyzer (Agilent). The Library Quantification Kit for Illumina (Kapa Biosciences) estimated the size and number of amplicon libraries. Samples were sequenced on the Illumina NovaSeq platform, and LC-Bio Technologies Ltd provided sequencing support. […] The raw reads were analysed using the Divisive Amplicon Denoising Algorithm 2 (DADA2) of the QIIME2 software. Quality filtering of raw reads is implemented by fqtrim software (V 0.94). DADA2 filtered the sequencing reads and output feature tags and sequences. Owing to the accuracy of the algorithm, fewer spurious sequences could be output. Species annotation was completed by BLAST, databases for comparison using SLIVA and NT-16 S. Alpha-diversity was described by the Chao1 index, Shannon index and Simpson index, with the P-value using the Wilcoxon test. β-diversity was performed by principal coordinates analysis (PCoA) based on weighted UniFrac distance metric and non-metric multidimensional scaling (NMDS) analysis using R ade4 (V 1.7–18) and vegan (V 2.5–7) package. The comparison of the relative abundance of relevant gut microorganisms between HCC patients (or ICC patients) and healthy controls was analysed using the Mann–Whitney test. […]"

<u>Deng et al. [47]:</u>

"Fecal samples were collected from each participant on enrollment and immediately frozen and stored in – 80 °C freezer for subsequent analysis. Gut bacterial taxonomy composition was detected by high-throughput V3-V4 region sequencing of the 16S rRNA in the fecal sample. The genomic DNA of each fecal sample was extracted using EZNA Stool DNA Kit (D4015, Omega, Inc., USA), which had shown to be effective for the preparation of DANN of most bacteria; the extracted DNA and subsequent elution with 50uL of Elution buffer were performed according to manufacturer's instructions and stored at – 80 °C for amplification. Next, PCR was performed to amplify the V3-V4 region of the 16S rRNA gene with primers 341F (5'-CCT ACG GGNGGC WGC AG-3') and 805R (5'-GAC TAC HVGGGTAT CTA ATC C-3') […], sample specified barcodes and sequencing universal primers was tagged at the 5' end of each primer. According to the manufacturer's instructions, PCR reactions were carried out with Thermo Scientific® Phusion High-Fidelity Hot start flex 2X Master Mix (New England Biolabs). The amplification system was constituted in 25 μL reaction system with 50 ng DNA template. The confirmation of PCR products was carried out by 2% agarose gel electrophoresis, and negative control of nuclear-free water was used to exclude the false-positive PCR results. The PCR products were purified by AMPure XT beads (Beckman Coulter Genomics, Danvers, MA, USA) and quantified by Qubit (Invitrogen, USA). The amplicon pools were prepared for sequencing, and the size and quantity of the amplicon library were assessed on Agilent 2100 Bioanalyzer (Agilent, USA) and with the Library Quantification Kit for Illumina (Kapa Biosciences, Woburn, MA, USA), respectively. The libraries were sequenced on the NovaSeq PE250 platform.

[…] Paired-end reads were assigned to samples based on their unique barcode and truncated by cutting off the barcode and primer sequence using cutadapt (version: 1.9) […]. Then, FLASH (version: 1.2.8) were used to merge paired-end reads within samples […]. Quality filtering and dechimerization were done by fqtrim (v0.94) and Vsearch software (v2.3.4) […]. Then, Divisive Amplicon Denoising Algorithm 2 (DADA2) […] in QIIME2 […] pipeline were used to denoise and dereplication the raw read and obtain the feature sequence and feature table of all samples. Compared to operating taxonomic units (OTUs) based analysis, DADA2 uses exact sequences to resolve the reads, enable more real variants, and output fewer spurious sequences than other methods. Next, the feature-classifier function in QIIME2 pipeline was used to align sequencing. Moreover, alpha diversity and

beta diversity were also conducted by QIIME2 pipeline, and the same number of sequences were randomly extracted from each sample to construct the relative abundance in bacteria taxonomy. The taxonomic analysis and species annotation were performed by BLAST, and the database used for taxonomy was SILVA (release 132) and NT-16S (2019.04.05). The results of sequences against SILVA were prior to NT-16S if the results were at odds. To screen the discriminatory taxa between groups, linear discriminant analysis effect size (LEfSe) […] was used to conduct high dimensional non-parametrical comparisons. Statistical Analysis of Metagenomics Profile (STAMP) […] was used to compare different abundance with Welch's-test. Principal coordinate analysis (PCoA) and nonmetric multidimensional scaling (NMDS) were performed to detect the distribution difference in taxa using Bray–Curtis dissimilarity and weighted UniFrac distance matrices. The statistically significant difference in α-diversities was analyzed by Kruskal–Wallis test and Wilcoxon test."

Jia et al. [48]:

"DNA extraction was carried out according to the MoBio PowerSoil DNA Isolation Kit 12888-100 protocol (Qiagen, Hilden, Germany), and DNA was stored at −80°C in Tris-EDTA buffer solution (Sigma-Aldrich, St. Louis, MO) before use. To enable amplification of the V4 region of the 16S ribosomal RNA gene and add barcode sequences, unique fusion primers were designed based on the universal primer set, 515F (5′-GTGYCAGCMGCCGCGGTAA-3′) and 806 R (5′-GGACTACNVGGGTWTCTAAT-3′), along with barcode sequences. PCR mixtures contained 1 μL of each forward and reverse primer (10 μM), 1 μL of template DNA, 4 μL of deoxyribonucleotide triphosphates (2.5 mM), 5 μL of 10 × EasyPfuBuffer (TransGen Biotech Co., Beijing, China), 1 μL of EasyPfu DNA Polymerase (2.5 U/μL), and 1 μL of double distilled water in a 50-μL reaction volume. Thermal cycling consisted of an initial denaturation step at 95°C for 5 minutes, followed by 30 cycles of denaturation at 94°C for 30 seconds, annealing at 60°C for 30 seconds, and extension at 72°C for 40 seconds, with a final extension step at 72°C for 4 minutes. Amplicons from each sample were run on an agarose gel. The expected band size for 515f-806r was about 300-350 bp. Amplicons were quantified with the Qubit dsDNA HS Assay Kit (Q32854; Thermo Fisher, Waltham, MA/Invitrogen, Carlsbad, CA) following the manufacturer's instructions. The amplicon library for high-throughput sequencing on the Illumina MiSeq platform was combined with an equal amount and subsequently quantified (KAPA Library Quantification Kit KK4824; Kapa Biosystems,Wilmington, MA) according to the manufacturer's instructions. […] Using the Quantitative Insights into Microbial Ecology 1.8.0 pipeline1, the raw sequences were processed to concatenate reads into tags according to the overlapping relationship; then, reads belonging to each sample were separated with barcodes, and low-quality reads were removed. The processed tags were clustered into the operational taxonomic units (OTUs) at the commonly used 97% similarity threshold. The OTUs were assigned to taxa by matching to the Greengenes database (Release 13.82). A phylogenetic tree of representative sequences was built. Alpha and beta diversity analyses were performed. Distances were calculated with R (3.3.1, flexmix package)."

Zhang et al [49]:

"All fecal samples from participants were freshly collected before treatment during the hospital stay and immediately frozen and stored in a −40°C freezer within 3 h of sampling (Wei et al., 2020). To reduce the effect of sampling bias, the middle portion of fecal matter was sampled in all cases. Bacterial genomic DNA was extracted using the EZNA® Stool DNA Kit (D4015, Omega, Inc., USA). The V3–V4 region of prokaryotic (bacterial and archaeal) small-subunit (16S) rDNA was amplified using slightly modified versions of the primers: 341F (5'-CCTAGGGNGGCWGCAG-3') and 805R (5'-GACTACHVGGGTATCTAATCC-3'). The DNA extraction process was carried out in ultrapure water to exclude the possibility offalse-positive PCR results. The PCR products were purified byAMPure XT

beads (Beckman Coulter Genomics, USA) and quantified using Qubit (Invitrogen, USA). The amplicon pools were prepared for sequencing using the Agilent 2100 Bioanalyzer (Agilent, USA), and Illumina's Library Quantification Kit (kappa Bioscience, USA) was used to evaluate the size and number of amplified sub-libraries. Samples were sequenced on an Illumina NovaSeq platform according to the manufacturer's recommendations, and the sequencing service was provided by LC-Bio Technology Co., Ltd., China. […] Raw reads were analyzed using QIIME2 software. Quality filtering of the rawreadswas performed using specific filtering conditions in fqtrim software (V.0.9.4) to obtain high-quality clean tags. Sequences with ≥100% similarity were assigned to the same feature. The DADA2 software was used to filter the sequencing reads and construct the feature table and sequences. As a result, the average reads were 63,191 (min = 39,815,max = 89,043). Sequence alignment for species annotationwas performed using BLAST, and the alignment database used was SILVA and NT-16S. Analysis of the dominant species in different groups and multiple sequence alignment was conducted using the MAFFT software (V.7.310). Alpha diversity of samples was described by the Chao1, observing species, goods_Coverage, Shannon, and Simpson indexes, which were calculated using Qiime2(2019.7), and P value was counted by Wilcoxon test. Beta diversity was calculated by principal coordinates analysis (PCoA) using R ade4 and vegan package."

Ito et al. [50]:

"Fecal samples were collected on the morning of the hospital visit, and a stool sample aliquot was mixed with 1 mL of guanidine thiocyanate (GuSCN) solution (TechnoSuruga Laboratory Co., Ltd., Shizuoka, Japan), immediately frozen at −80 °C, and stored until analysis. DNA extraction from the human fecal samples was performed using the bead-beating method as previously described, with some modifications […]. Briefly, 150 μL of fecal sample in GuSCN solution was vigorously vortexed with 300 mg of glass beads (AS ONE BZ-01) and 500 μL of Tris-EDTA (TE, pH 9.0) buffer-saturated phenol (Fujifilm, Wako Pure Chemicals) using a FastPrep-24 (Funakoshi Corporation) for 30 s at power level 5. After centrifugation at 10,000× g for 10 min, 400 μL of the supernatant was extracted with 500 μL of phenol–chloroform, and 250 μL of the supernatant was precipitated with isopropanol. The purified DNA was suspended in 100 μL of TE buffer (pH 8.0). […] Amplicon sequencing of the V3-V4 regions of the bacterial 16S rRNA gene was performed with an Illumina MiSeq instrument, as described previously […]. Data were analyzed in the QIIME2 software package […] (ver. 2017.10). The reads were mapped to the PhiX 174 sequence and the Genome Reference Consortium human build 38 (GRCh38) by the Bowtie-2 program […] (ver. 2–2.2.4), and potential chimeric sequences were removed from acquiring the Illumina paired-end reads by using DADA2 […]. Thereafter, 30 and 90 bases of the 3′ region of the forward and reverse reads were trimmed, respectively. Taxonomic classification was performed using a naive Bayes classifier trained on Greengenes 13.8 […] with a 99% threshold for operational taxonomic unit (OTU) full-length sequences. An estimation of alpha diversity and a principal coordinate analysis (PCoA) for beta diversity were also performed using QIIME2."

Zhang et al. [51]:

"A fecal sample was obtained by the participants, transported to the lab within 2 h, and stored at-80°C until DNA was extracted. The CTAB method was used to extract DNA, and 1% agarose gels were used to measure the DNA concentration. The hypervariable ITS2 region of the ITS gene was amplified using the primers [ITS3-2024F (5′-GCATCGATGAAGAACGCAGC-3′) and ITS4-2409R (5′-TCCTCCGCTTATTGATATGC-3′)] with the barcode. Mix the same volume of 1 x loading buffer (containing SYB green) with the PCR products and operate electrophoresis on 2% agarose gels for detection. The PCR products in the mixture were then purified using Thermo Scientific's GeneJET Gel Extraction Kit. Using the TruSeq® DNA PCR-Free Sample Preparation Kit (Illumina, United States), sequencing libraries were made according to the manufacturer's instructions, and index codes were

added. To evaluate the library's quality, Thermo Scientific's Qubit@ 2.0 Fluorometer was used. Finally, the library was sequenced, and 250 bp paired-end reads were produced on an Illumina Novaseq 6,000 PE250 platform. The NCBI Sequence Read Archive database contained the raw sequencing information for all samples (PRJNA898483). Paired-end reads were assigned to samples according to their unique barcode and truncated by cutting off the primer sequence and barcode. Then, FLASH (V1.2.7)1 was utilized to merge paired-end reads […]. Quality filtering on the raw tags was conducted to obtain the high-quality clean tags […] based on the QIIME (V1.9.1)2 quality-controlled process […]. Clean Tags were compared with the Unite Database3 to detect chimera sequences, and then the chimera sequences were removed. Using the Uparse software (Uparse v7.0.1001;4 Edgar, 2013) to cluster all Effective Tags, the sequences with ≥97% similarity were assigned to the same OTUs (Operational Taxonomic Units). The sequence with the highest frequency of occurrence in OTUs was chosen as the representative sequence of OTUs. For each representative sequence, the Unite Database […] was used based on the blast algorithm to annotate taxonomic information […]. A Venn diagram was produced via the R package "VennDiagram" (Version 2.15.3) to show the shared and unique OTUs between the two groups. Using R software (Version 2.15.3), the rank abundance curves were presented. Using QIIME (Version 1.9.1), the alpha diversity (Shannon, Simpson, Chao1, and ACE) and the beta diversity on weighted unifrac distances were determined. The non-metric multidimensional scaling (NMDS) analysis was conducted using the vegan package of R software […], and the principal co-ordinates analysis (PCoA) analysis was completed using the WGCNA, stats, and ggplot2 packages of R software […]. The R package vegan's MRPP function was used for multiple response permutation procedure (MRPP) analysis […]. The linear discriminant analysis effect size (LEfSe) analysis was carried out using the LEfSe software (Version 1.0) with a default setting of 4 for the linear discriminant analysis (LDA) score screening […]. Spearman analysis of the top 20 species in abundance at the genus level with clinical parameters of ICC patients. To further explore the impact of mycobiota community change, functional prediction with FUNGuild annotation tools was deployed (A NHN, B ZS, B STB, 2016)."

<u>Mao et al. [52]:</u>

Baseline fecal samples were collected at the starting point of anti-PD-1 treatment and dynamic fecal samples were continuously collected the day before each anti-PD-1 monobody infusion every 3 weeks. All fresh stool samples were stored in sterile containers and frozen at −80°C within 24 hours until DNA extraction. [...] Bacterial genomic DNA was extracted using a cetyltrimethylammonium bromide kit according to the manufacturer's instructions. The DNA concentration and integrity were checked to screen qualified samples for sequencing. Individual libraries were constructed using the NEBNext Ultra DNA Library Prep Kit and DNA sequencing was performed on the Illumina NovaSeq 6000 platform using a 2×150 bp paired-end read protocol. Raw data were filtered with Trimmomatic to obtain high-quality clean reads. The host DNA sequence was removed by aligning to the Homo sapiens genome assembly hg38 with Bowtie 2 to obtain metagenomic DNA sequences. The metagenomic DNA sequences were assigned taxonomic labels using the Kraken 2 program and then Bracken (Bayesian Reestimation of Abundance after Classification with KrakEN) was used to estimate the abundance of each sample at different phylogenetic levels (phylum, class, order, family, genus, and specie). The quantitative metagenomics analysis was finished by Wekemo Tech Group Co., Ltd. (Shenzhen, China). For phylogenetic diversity between the CBR group and NCB group, alpha diversity was computed using the R package vegan to evaluate the richness and evenness of each sample, and then compared with the Wilcoxon test. Beta diversity based on Bray-Curtis metrics was applied to compare the dissimilarities between different groups with principal coordinate analysis (PCoA). Common or unique taxa at each phylogenetic level between different groups were visualized using the R package VennDiagram. Based on the relative abundance, significantly differential taxa between the CBR group and NCB group were identified with the Wilcoxon test, and an alluvial

diagram was used to show the association among different phylogenetic levels for the genus or species using the R package ggalluvial. Linear discriminant analysis (LDA) effect size (LEfSe) was further applied to identify significantly differentially enriched taxa between the CBR group and NCB group. […] The metagenomic DNA sequences were aligned against the UniRef90 database using the DIAMOND algorithm and then the HUMAnN2 tiered search strategy was used to quantify the KEGG Orthologous (KO) groups abundance against the Kyoto Encyclopedia of Genes and Genomes (KEGG) database. Significantly differential KOs between the CBR group and NCB group were identified by LEfSe and potential functional species in different KOs were also identified. Metabolic pathways were further verified in the MetaCyc database."

Jin et al. [53]:

"Bacterial DNA was extracted from patients' fecal specimens using the QIAamp DNA Stool mini kit (QIAGEN). The hypervariable regions (V3–V4) of the 16 S rRNA gene received amplification and sequence on the HiSeq Illumina platform. Raw reads were analyzed using USEARCH software (version 11) for quality control, operational taxonomic units (OTU) clustering, and taxonomy annotation. All pairs of sequences with identity ≥97% were distributed to the same OUT. The representative sequence for each operational taxonomic units was screened for further annotation with the Silva rRNA gene database release based on the RDP classifier algorithm. MetaPhlAn2 was used for microbial taxonomic profiling, including the relative abundance information of each specimen in each hierarchy (kingdom, phylum, class, order, family, genus and species). Analyzed by R/Bioconductor packages, nonlinear unsupervised clustering was used to identify microbiome configurations with distinct responses to study treatment. Microbiota relative abundance was visualized by heatmap, in which the cluster_rows and cluster_columns were specified to a hclust or dendrogram object. The potential microbiome biomarkers in relation to clinical outcomes were determined at the species level. The sequencing coverage and quality statistics for each sample are summarized in Supplemental Table S2."

Zhang et al. [54]:

"DNA extraction and amplification were performed using Eppendorf liquid handling robots. The V4 region of the 16S rDNA gene (515F-806R) was sequenced for 10 samples for Fig 1A, and 15 samples for Fig 2G; generating paired-end, overlapping reads on the Illumina MiSeq platform […]. The demultiplexed paired end fastq files were pre-processed and analyzed using QIIME 2 version 2–2020.2 (https://qiime2.org) […]. The DADA2 algorithm […], implemented in QIIME2, was used for error modelling and filtering the raw fastq files. Post denoising and chimera removal; a total of 573,739 sequences was retained for 10 samples with an average of 57,373 sequences per sample for Fig 1A, a total of 835,408 sequences was retained for 15 samples with an average of 55,693 sequences per sample for Fig 2G. Taxonomic classification was performed using the QIIME2 feature-classifier (https://github.com/qiime2/q2-feature-classifier) plugin trained on the Silva 132 database […]. The Alpha and Beta-diversity analyses were performed using the diversity plugin (https://github.com/qiime2/q2-diversity) at a rarefied sampling depth of 50000 for Fig 1A, and at a rarefied sampling depth of 49700 for Fig 2G. The Bioproject accession number of the 16s rRNA sequencing for mouse stools is PRJNA680370. […] DNA extraction and 16s rDNA sequencing for human samples (Supplementary Fig. S9A) were performed as described […]. Raw 16s rRNA sequencing reads of human stool samples can be found in the NCBI SRA associated with Bioproject PRJNA517994 (Cirrhosis) and PRJNA525701 (Healthy), under the following BioSample IDs: SAMN11083186 (Healthy), SAMN10856936 (Cirrhosis #1) and SAMN10856982 (Cirrhosis #2)."
